# Harm reduction social work with people who use drugs: a qualitative interview study with social workers in harm reduction services in Sweden

**DOI:** 10.1186/s12954-023-00884-w

**Published:** 2023-10-14

**Authors:** Torkel Richert, Anke Stallwitz, Johan Nordgren

**Affiliations:** 1 Department of Social Work, Malmö University, Nordenskiöldsgatan 1, 211 19 Malmö, Sweden; 2 Department of Social Work, Protestant University of Applied Sciences Freiburg, Bugginger Straße 38, 79114 Freiburg, Germany

**Keywords:** Drug addiction, Harm reduction, Social work, Harm reduction social work, People who use drugs, Rehabilitation, Recovery

## Abstract

**Background:**

Social work with people who use drugs (PWUD) has traditionally focused on abstinence and rehabilitation. In recent years, harm reduction has gained an increasingly more important role in social work with PWUD, and social workers are key professionals in many harm reduction services. This study investigates how social workers in harm reduction services for PWUD in Sweden understand the concept of harm reduction and how it relates to goals of rehabilitation, and how they assess and deal with dilemmas and challenges in everyday work.

**Methods:**

The study is based on interviews with 22 social workers in harm reduction services for PWUD in the Scania region of Sweden. A thematic analysis in three steps was used in coding and processing the data.

**Results:**

The social workers pointed to similar values between social work and harm reduction and argued for combining the two fields to improve services for PWUD. Three overarching principles for Harm Reduction Social Work (HRSW) were developed based on the social workers accounts: (1) Harm reduction is a prerequisite for rather than a counterpoint to rehabilitation and recovery, (2) motivational work must be non-mandatory and based on the client’s goals, (3) a holistic perspective is crucial for Harm Reduction Social Work. Challenges in doing HRSW concerned restrictive laws, policies, and guidelines, resistance from managers, difficulties in setting boundaries between client autonomy and life-saving interventions, and the risk of normalizing high-risk behaviors.

**Conclusions:**

We use the concept of Harm Reduction Social Work to show how social work with PWUD can have a primary focus on reducing harm and risks, while at the same time it involves a holistic perspective that facilitates motivation and change. The suggested principles of HRSW can provide guidance in practical social work with vulnerable PWUD. Social workers can have important roles in most harm reduction settings and may act to enable recovery.

## Introduction

Social work with people who use drugs (PWUD) has traditionally focused on abstinence and rehabilitation with the goal of helping clients to stop using drugs, change their lifestyle and be reintegrated into society [1, 2]. In recent years, harm reduction has gradually entered social work discourse and practice and is now seen as a promising approach for helping and treating individuals with drug and alcohol problems [3]. Harm reduction refers to a broad set of goals, strategies, and services which aim to minimize the social and physical harms of substance use, without necessarily aiming for abstinence. Important harm reduction services for PWUD include needle and syringe exchange programs, overdose prevention, drug consumption rooms, opioid substitution treatment (OST), low-threshold housing, and drug checking [4].

Harm reduction approaches have been described as an alternative to the moral and disease models of addiction that have dominated substance use treatment, and as a means of reducing problematic power dynamics in social work practice [3]. Social work, especially that which is carried out by employees within state or municipal authorities, includes to varying extents a power hierarchy of social worker over client, as well as practices of surveillance and control [5]. Proponents of a harm reduction perspective in social work with PWUD argue that this could reduce moralism, imply a reduced focus on abstinence, lower thresholds, and increase equality in the relationship between client and professional [1, 6–8].

On the other hand, social workers and social science researchers have raised concerns about what too strong or narrow a focus on harm reduction in the work with PWUD could lead to. The development of harm reduction has been associated with individualization and medicalization of the drug problem, and with addressing short-term issues and symptoms of complex problems, rather than the broader social, political, and economic context that creates these problems [9, 10]. This also relates to the risk of health-related goals being prioritized over goals such as social integration and rehabilitation [11]. Critics have also argued that harm reduction can be a cynical strategy that shows too little faith in people’s ability to change [12], that harm reduction practitioners accept the client’s engagement in high-risk substance use rather than imposing the safest alternative: abstinence [13], and that harm reduction can imply fatalism in the form of a “palliative care model” [9].

A further area of controversy between those who advocate for total abstinence and harm reductionists is the conceptualization of recovery. The recovery-based treatment system, in which social workers can have important roles, is traditionally oriented toward achieving complete abstinence among clients, responsibility and a drug free society. This stands in contrast to the harm reduction perspective which focuses on the client’s own goals without demanding abstinence or lifestyle change. However, more recent definitions of recovery incline toward inclusiveness, well-being, and improved quality of life and acknowledges that recovery is something that is made in practice and can take multiple forms [14]. The introduction of the concept recovery capital has meant a stronger focus on the individuals’ different strengths and needs and on community-based recovery sources. Recovery capital usually involves the sum of the individual’s social, physical, human, and cultural capital that can be decisive in the initiation and maintenance of substance misuse cessation [15]. A resent proposal of an assemblage approach to recovery highlights that recovery should not be seen as a separate, post-drug use phase, but rather should be treated as part of drug use and in the relation to drug use [14]. These developments show that recovery can have multiple meanings and implications that may conflict or harmonize with a harm reduction perspective. Lancaster and colleagues state that the polarization of the field between harm reduction and abstinence-based approaches in Britain, and a shift toward a recovery-oriented drug policy has created a concern about how this might affect the continued provision of harm reduction [16].

Although there are some dividing lines between social work and harm reduction, the two fields also share many core values, and could be combined to provide a more comprehensive continuum of services [1]. The harm reduction model has even been put forward as an ideal framework for social work practice in a wide variety of settings and as something that should be a fully integrated component of social work education [2]. Vakharia and Little [3] state that harm reduction and social work are “natural partners” with similar core values, including a client-centered and strengths-based approach, and a focus on developing a working alliance and supporting the client’s self-efficacy. Given these similar values they believe that “*it is only a matter of integrating the specific framework and treatment interventions for social workers to be leaders in harm reduction practice”* [3, p 67].

Even though harm reduction increasingly has been suggested as a constructive perspective for social work, few guidelines for clinical practice have been detailed in the social work literature, something that limits the potential implementation of the model into day-to-day social work practice [3]. There is little knowledge about social workers’ roles in harm reduction settings, and about what they see as the dilemmas and advantages of doing social work with a focus on harm reduction**.** There is also a lack of knowledge about what social workers, or a social work perspective, contribute to harm reduction services.

This study aims to investigate how professional social workers in harm reduction services for PWUD understand the concept of harm reduction and how the concept relates to rehabilitation, as well as how they assess and deal with various dilemmas and challenges in everyday work with PWUD. The study is based on semi-structured interviews with 22 social workers in harm reduction services for PWUD in the Scania region of Sweden.

## Social work and harm reduction in Sweden

In Sweden, healthcare and social services have a shared responsibility for treatment and support for PWUD. The healthcare sector primarily provides medically oriented efforts, while social services are responsible for non-medical treatment, accommodation, social support, and rehabilitation as well as the investigation of care needs of PWUD. Social workers are represented in most services for PWUD, including harm reduction programs such as OST, housing, and needle and syringe exchange programs. Social workers are thus one of several key actors in the work with PWUD in Sweden, and the social work perspective has been important in the development of drug policy and interventions within the addiction field [17].

Since the 1980s, Swedish drug policy has been based on a zero-tolerance approach with the stated aim of creating a “drug-free society” [18]. Although the strategy has been built on three pillars—control, prevention, and treatment—a proportionally large focus has been placed on various control efforts [19]. Historically, harm reduction has developed relatively slowly in Sweden since interventions primarily aimed at reducing risks and harms of drug use, without the goal or requirement of abstinence, have been considered controversial. When introduced, harm reduction services have been met with resistance and have been forced to incorporate strict regulations and controls as well as, in some cases, requirements for motivational work toward treatment or abstinence [20, 21]. This has led to some PWUD being excluded from OST or choosing not to start treatment, instead self-medicating with illegal substances [22]. Social workers have historically had an active role in the resistance toward harm reduction. For example, both OST and needle and syringe exchange programs were initially opposed by individuals in the social work profession [23–25]. In the last decade, however, the harm reduction perspective has gained ground, and existing harm reduction services have expanded and become less regulated, and new services have been introduced. Access to OST has gradually increased, the number of syringe exchange programs has increased significantly, and take-home naloxone programs have been initiated and developed in most regions [19, 26, 27]. Housing first programs and low-threshold housing with an acceptance of drug use have been introduced in some municipalities. However, interventions such as heroin-assisted treatment, drug consumption rooms, and drug testing have still not been introduced in any regions in Sweden [19, 28].

Harm reduction measures have been geographically unevenly developed in the country [29]. This is also reflected in social workers’ attitudes. Although social workers now generally tend to have positive attitudes toward harm reduction, social workers in regions with low access to services tend to be more negative toward harm reduction goals and specific services in comparison with social workers in regions with high access [30]. Scania, Sweden’s most southerly region and the location for this study, stands out when it comes to harm reduction services for PWUD. Two syringe exchange programs started as pilot projects in 1986 and 1987, 20 years before a new law made it possible to start programs in other parts of the country. Scania was also one of the first regions to implement a take-home naloxone project [26] and was the first region in the country to implement a choice of care for OST, which led to increased accessibility and freedom of choice for people with an opioid addiction [27]. In Malmö, the largest city of the region, the first low-threshold accommodation in Sweden with a clear harm reduction focus was started, allowing residents to use illegal drugs in their rooms. A recent study from Malmö [31] showed that police officers largely supported harm reduction services in the city and refrained from enforcing drug laws in their vicinity.

Many of the harm reduction services and initiatives in the region have broken new ground and were initially perceived as controversial. Social workers are employed in all the services, and many of them have also been active in pushing forward the boundaries of harm reduction. This is interesting and points to a possible shift in the focus of social work, since addiction-related interventions from the social services in Sweden have traditionally had abstinence and social integration as their main goals [32]. These goals are also emphasized in the Social Services Act (Chapter 5, section 9), which states that municipal social services have an obligation to ensure that *“the individual addict receives the help and care he or she needs to recover from the addiction”*.

The Swedish case, where social workers have a central role in addiction care and rehabilitation in general and in harm reduction services specifically, can provide important insights into experiences of and challenges for social workers in harm reduction settings, and about what a social work perspective can contribute in harm reduction services.

## Methods

### Recruitment and sample

We conducted semi-structured in-depth interviews with 22 social workers employed at harm reduction services for PWUD in Scania County in the south of Sweden. The interviewees were recruited through a purposive sampling approach [33], aiming to reach a broad group of social workers in terms of age, gender, number of working years and different types of workplaces and work tasks. Our long-standing contacts with services for PWUD in the region facilitated this recruitment process. For several years, we have led a research study circle with social workers who work with PWUD in Malmö. This means that we have good contact with staff and managers from various low-threshold operations in the city. Several circle participants were asked if they themselves wanted to participate in the study and if they had suggestions for other possible participants.

The interviewees were recruited on the basis that they were professional social workers working within harm reduction services for PWUD within the region. The services included low-threshold accommodations where drug use is permitted, OST clinics, needle exchange programs, an outreach team for homeless PWUD, and an NGO providing food, social activities, and health services for PWUD and people experiencing homelessness.

Eighteen of the interviewees were female and four were male. All interviewees were professional social workers, which in Sweden means that they have completed a bachelor’s degree in social work of at least 3.5 years. Their average age was 40.5 years, with a range between 25 and 62. The median number of years of practice as social workers was 12 years, with a range between one and 31 years.

### Interviews

Due to the Covid-19 pandemic, the interviews were conducted in two waves, one in 2019 and one in 2022. We conducted the interviews face to face at the interviewees’ respective workplaces. The majority of the interviews were conducted individually, but in two cases they were conducted as pair-interviews at the request of the interviewees. The interviews were recorded, and they lasted between 40 and 110 min.

All interviewees gave informed oral and written consent to take part in the study. To ensure the anonymity of the interviewees, we have changed their names and anonymized their organizations.

We used a semi-structured interview guide to allow a focused conversation about the interviewees’ practices, experiences, and attitudes. The interview guide contained sections about; (a) background information (age, gender, educational background), (b) their views of drug use problems, drug scenes and services for PWUD in the region, (c) assessments of PWUDs’ vulnerability and the possibility of receiving help, (d) professional role and work situation, (e) opinions of Swedish drug policy, (f) views on conflicts and similarities between social work and harm reduction, (g) possibilities and risks in conducting motivational work, (h) views on challenges and opportunities in their harm reduction work. In this paper, we focus primarily on responses from sections d, e, f, g and h.

### Analysis

The interviews were transcribed verbatim by a research assistant and checked for accuracy by the first author. Our approach to analyzing the empirical material was based on qualitative text analysis and aimed at interpreting meaning from the empirical material [34]. Coding was carried out by the first author in a three-step process, influenced by Braun and Clarke’s thematic analysis [35]. The first step consisted of reading the transcribed interviews, with the aim of obtaining a holistic view of the material. In the next step, the material was categorized into broad themes relating to the overall focus of the study such as “similarities and differences between social work and harm reduction”, “difficulties and dilemmas in doing harm reduction work”, “what social work can contribute within harm reduction services”. In a later stage, “Harm Reduction Social Work” gradually emerged as a core theme. In further analysis of the data, the overall themes were categorized into more specific subthemes. In the third step, quotations that represented the identified themes were chosen as analytical points and interpreted for meaning. The interview excerpts were translated from Swedish to English by the first author and then checked by a professional proofreader to ensure that the meaning of the quotations was retained.

## Results

### Harm reduction social work: principles and strategies

The interviewed social workers all stated that harm reduction should be a natural part of social work with PWUD. They believed that there are great similarities in the basic ideology behind harm reduction and social work, such as the importance of “*meeting the person with respect and dignity*”, “*meeting the person where they are at*” and “*starting from the client’s own goals*”. At the same time, some of the social workers pointed out that their work differed from what they called “traditional social work” which includes a stronger focus on long-term abstinence, rehabilitation, and self-sufficiency, but also differed from more medically oriented harm reduction work which they believed could lack a holistic perspective and social dimensions.

The social workers saw great advantages in their work being oriented toward harm reduction and at the same time believed that as social workers they could add important dimensions to the harm reduction perspective. Their descriptions can be summarized in three overarching principles and strategies where harm reduction and social work can be combined in what we define as Harm Reduction Social Work (HRSW).

#### Harm reduction is a prerequisite for rather than a counterpoint to rehabilitation and recovery

The social workers emphasized that harm reduction must come first and permeate the work with vulnerable PWUD. This was motivated by the fact that for most people, lifestyle change is only possible if they have a basic level of security, somewhat stable health, and a reasonably stable social situation. At the same time, they pointed out that harm reduction does not oppose motivational work or rehabilitation and that social workers have an important function in making possible alternatives and room for action visible to clients.

Marcus, a social worker in low-threshold housing, clarified their priorities: *The focus of our service is harm minimization, so we have the three directions: number one is to save lives by offering a safe place, number two is to minimize harm, and number three is to help the client further in the direction the person wants.*

When asked how a social worker in a low-threshold service should relate to the overarching political goal of a drug-free society and to the Social Services Act’s stated goal of helping people with addiction stop using drugs and rehabilitate, Peter, a supervisor at a low-threshold residence, answered:

A drug-free society will never happen anyway, so you can just drop that goal, that’s what makes it [the drug policy] so repressive. The second part, helping to stop [using drugs], yes, but to be able to stop, you have to be alive and the less harm you get from the use, the better chance you have of coming back. That is the kind of harm reduction that we do here, we give people the energy to be able to make a choice. So, if you sleep, eat, have an ID card, if you have your medicines, yes, but then you can at least start thinking about “Do I want to live like this or do I want to reduce…” If you are on the street and are being chased [by the police], never have money and inject as many drugs as you can just to last another day, yeah but then, that's no help to stop using.

The quote points out that a repressive drug policy can increase vulnerability and risks for PWUD and reduce the possibilities for rehabilitation. In contrast, according to Peter, a harm reduction approach can reduce suffering and increase the chances for the person to be able to decrease drug use and change their life situation in the long term. In this sense harm reduction is a prerequisite for, rather than a counterpoint to, rehabilitation and recovery.

The interviewees said that with most people they could work in parallel with harm reduction and gradual rehabilitation. In some cases, however, the work was almost exclusively about keeping the person alive and reducing risks as much as possible. The social workers argued for a broad concept of harm reduction that included for instance: treating wounds or injuries, efforts to improve physical and mental health, working with strategies to reduce exposure to violence or sexual abuse, counteracting loneliness, improving sexual health, working with overdose prevention, educating in safer ways to use drugs, and offering food, clothing and a safe place to sleep.

#### Motivational work must be non-mandatory and based on the client’s goals

A harm reduction perspective—where you meet individuals where they are at, without demands for abstinence or lifestyle change—was described as crucial for creating a trusting relationship and for being able to carry out motivational work. Hanna, who worked at a low-threshold housing unit, talked about the great advantages of working primarily from a harm reduction approach:

You increase the chance of building an alliance and trust in the person. There are no controls or any control function that make the person in question need to deceive, lie, manipulate… also by showing that I want to help you regardless of what you want help with, regardless of whether you choose to use drugs or not, I’m here to help you and care about you, I want to listen to what you have to say.

The quote shows the importance of not making unreasonable demands or controlling clients and of basing the work on the client's goals. According to Hanna, this is crucial for a trusting and honest relationship and for the possibility of conducting motivational work.

Another central theme from the interviews was that motivational work can never be imposed. Moa, a social worker who did outreach work with socially vulnerable PWUD, said the following about the opportunity to work with motivation for change:

We definitely engage in motivational work and try to motivate them to dare to seek help, but then it is more if the person wants but maybe doesn't dare. But we don’t have goals for our clients; they have to set them themselves, so in that way there isn’t much work toward change if the person doesn’t initiate it themselves. And sometimes there really isn’t any change-work at all, but just making sure the person doesn’t perish.

The quote points out that working toward change or recovery is not always possible. At the same time, it shows the importance of analyzing possible obstacles to clients’ motivation and change. It was considered common that clients themselves did not believe that change was possible, that they did not dare to seek help or “open up” due to feelings of shame or the risk of being poorly treated or met with prejudice. Helping clients to overcome these obstacles and making visible alternative courses of action were seen as important tasks for social work with PWUD.

Pia, a social worker in a low-threshold housing unit, described how staff working close to PWUD with great vulnerability develop a different perspective on what constitutes positive change or success. Changes could involve the client switching from a more dangerous drug to a less dangerous one, injecting with sterile equipment instead of used, starting to eat more regularly, or making an initial contact with health care or psychiatric services. These types of changes were considered major successes by the staff but were rarely seen as decisive by outsiders. Pia said: “*We see changes all the time, which we think are good for the client in the long run but which society may not appreciate.”* She further explained that from society’s perspective, everything about addiction is black or white, *“Is he an addict or a non-addict*, *everything in between doesn’t matter”.*

According to the interviewed social workers, harm-reducing social work must be based on an understanding that lifestyle change is not always possible or desirable, that it can take a long time, and that even small changes can be of great significance. The general view was that professionals must be sensitive to their clients’ motivation to change and facilitate this change without being intrusive.

#### A holistic perspective is crucial for harm reduction social work

Something that was stated in many interviews was that a holistic perspective should be a starting point for harm reduction social work with PWUD. Although all social workers agreed that harm reduction is part of social work with PWUD, several said that social work in some respects can also be said to be *“more than harm reduction”*, that social work with PWUD means something *“in addition to reducing harm and vulnerability”*. A holistic approach was described as seeing the individual in a larger context, analyzing possible underlying causes of the drug addiction, acknowledging the individual's strengths, enabling enjoyment and pleasure, and involved counteracting structural obstacles to change for the individual. Several social workers believed that this holistic perspective could add important dimensions to what was, in some cases, a rather narrow or medical harm reduction perspective.

Anna, who worked in a low-threshold housing unit, developed the notion of what a holistic perspective can mean when working with PWUD.

Many people have the opinion that you have to fix the problem [drug abuse] first and then you can work on other things such as rehabilitation and strengthening different life areas. But you can turn the tables and do exactly the opposite, you focus on everything else so that the drug use does not become as important anymore. So that it gets less space or less focus, so that other things in life become more positive, so that you either cut back or stop using, or stop using a certain drug, maybe change social interactions, make new friends.

Anna pointed to the importance of looking beyond drug addiction and the consequences connected to this and instead focusing on the individual as a person. By strengthening the individual’s resources, skills, and networks, Anna believed that the individual’s opportunities to see other possibilities for action become greater. The “drug-free life” must, according to Anna, offer something that replaces not only the psychological functions of the drug but also the social life, income strategies, and skills that have been linked to a lifestyle where the person organizes their everyday life and interactions largely around drugs.

An additional dimension of a holistic perspective highlighted was mapping which resources, networks, and services the person might need. Some described this as *“being the spider in the web”* or *“building bridges”* to different services. It could be about accompanying the person to a doctor’s visit, to the social services or to the employment agency, or about making contact with a user organization or activity center. Bridge-building was considered particularly important when working with PWUD who had limited social abilities or very low trust in authorities. Hanna, a social worker at one of the low-threshold housing units in the city, described this work in terms of being a “middle ground”:

And the biggest gain [of a harm reduction approach] is that you can often motivate the person to accept help from others as well. You can be a bit of a middle ground, between different [services], yes, but if they have confidence in me, if I say it’s fine to go to this doctor, we can go to that doctor together. And also, if any problems arise in the contact with this doctor, you can stand up for the person and so on.

In other words, a holistic perspective also involved helping the client navigate a help system that can sometimes be bureaucratic and difficult to access; it was about mediating contacts and, in some cases, about standing up for the client's social and human rights.

The holistic perspective relates to a broad perspective on harm reduction, which can be about reducing risks and vulnerability in many different areas of life, including medical, psychological, and social aspects.

In more medically oriented services such as OST and needle and syringe exchange programs, social workers were considered to have a particularly important role in ensuring that the holistic perspective was represented. It could involve having time for more *“in-depth conversations where you can process various problems”*, to focusing on *“social network, housing and other issues in life”* in addition to illnesses, health problems, and medications. Without this broadened focus, there is a risk that, OST clinics, as Malin expressed it, *“only become a place where you get medicine without the possibility of additional support”*.

### Challenges in doing harm reduction social work

The interviewed social workers all talked about challenges in doing HRSW with PWUD. The most important challenges can be summarized in two overarching themes: (1) professional, organizational, and policy limitations, (2) setting boundaries and dealing with normalization of risk.

#### Professional, organizational, and policy limitations

At a policy level, legislation and guidelines were described as obstacles to carrying out or developing harm reduction work. In general, harm reduction services in Sweden have had high requirements for enrollment, clear goals for motivational work, strict rules and low thresholds for dischargement. This has gradually changed over the past 10–15 years, although some restrictive rules and control efforts remain. Examples of policy level limitations that were raised concerned legal barriers to handing out syringes (except in needle and syringe exchange programs) or implementing safe consumption rooms, laws that means needle and syringe exchange programs have age limits and that require visitors to show identification, and legal barriers to social workers being allowed to carry naloxone.

Another example that was raised was that certain harm reduction services have guidelines or legal requirements that the staff must carry out motivational work. Although a focus on motivational work was generally seen as positive, it was believed that stated requirements for this could involve problems. Fredrik, a counselor at a syringe exchange program saw the risk that social workers would *“force motivational talk on clients”* as relatively small, but at the same time pointed to problems with this type of statutory requirement:

If you are exposed to motivational work when you do not want it then it contributes to a feeling of stigmatization that you often may already have. I can imagine that this can be very difficult for the patients. It is difficult to know, but perhaps there is such a risk [to force motivational talk on clients] if it says in the legislation that you must do it [motivational work], if you then interpret it literally, then perhaps there is such a risk. Then you probably lose some patients because of that.

On an organizational level, limitations could entail resistance from the higher management in being allowed to push harm reduction as far as they wanted, not least within municipally organized services. Examples were a stalled initiative to establish “locker rooms” where homeless PWUD could store belongings, and a resistance to publishing brochures with information on safer injecting practices and overdose prevention at a low-threshold accommodation. Many of the interviewed social workers had themselves pushed for further development of harm reduction in the city, despite opposition.

On a professional level, limitations concerned unreasonable expectations and demands placed on the social work with PWUD from colleagues in social services. Svea, who worked as a counselor at an OST clinic, believed that some social workers who had “their” clients at the clinic had a poor understanding of the client’s situation and unreasonable expectations of lifestyle change. This included, for example, the expectation of *“total abstinence from illegal drugs”* or a clearer *“focus on employment and rehabilitation”* in the work with the clients:

Some social workers can push this issue quite far. And be assertive about it, like—‘how can you allow my client to be so intoxicated?’ And so on. They have somehow not understood that this is a long process that can take up to two years, perhaps, before a person becomes stable.

A few social workers also talked about clients having expectations that meetings with social workers must involve talking about motivation to change, something that could negatively affect the relationship. Fredrik, a counselor at a syringe exchange program, said that some patients experienced ambivalence about contacting him because he was a social worker “*Why should I see a counselor, I have no plans to stop using drugs”.* He further stated that “*there is still a notion among some drug users that there is an expectation from us [social workers] that the purpose of the contact is for them to quit [using drugs]”.*

Another challenge related to professionality concerned social workers lacking the medical competence or legitimacy required for certain harm reduction tasks. In some services, for example a residence for PWUD, all the staff were social workers. This meant that they experienced certain limitations in their daily work, not being able to handle prescriptions, medication, or serious medical injuries. The staff also did not have the legal right to carry naloxone, despite overdoses occurring among their clients. Some of these challenges could be solved through close cooperation with health centers or the syringe exchange program and through the residents themselves sharing naloxone with the staff. In services with only one or two social workers, such as syringe exchange programs or OST clinics, they could sometimes feel alone in representing a social perspective within a more medical context.

#### Setting boundaries and dealing with normalization of risk

The social workers agreed that harm reduction interventions for PWUD should have *“low thresholds in*, *high thresholds out”* and have as few rules as possible. At the same time, certain rules and restrictions were considered necessary for protecting clients from themselves or from each other, for protecting staff, or for protecting the reputation and legitimacy of the services. The social workers presented several examples of ethical dilemmas and difficulties around setting boundaries in the work.

One example concerned client inclusion criteria. This is partly regulated in laws and guidelines, but services also set their own boundaries. For example, some accommodations and low-threshold services did not enroll young people or people early in their ‘addiction career’ because it was considered that there was a risk of their situation worsening. However, it was seen as difficult to determine what *“young”* and *“early in the addiction career”* meant and what the consequences would be for PWUD who were not enrolled.

Other boundaries concerned when to discharge clients or when to make a report of concern for compulsory care (something that social services in Sweden can suggest if the drug addiction poses a life-threatening danger to the individual), or even call the police. In general, it was agreed that these measures should be avoided as far as possible and that there must be a high level of acceptance for risky behavior, rule-breaking and “disorderliness”, considering that most clients had a long-term drug addiction in combination with mental illness. In addition, there were few other options for the clients, and a discharge from, for example, a low-threshold housing unit or OST clinic would very likely mean that the person ended up in a worsened life situation with increased exposure to risk.

Examples of situations that were considered difficult to handle were when patients were psychotic or aggressive, when they had repeated overdoses or life-threatening health problems, and when they sold drugs in or in connection with the services. In some cases, especially concerning aggressiveness or violence directed at other clients or staff, patients could be discharged from the service or referred to another agency. In general, however, very few violent incidents were described. Drug sales were not allowed at OST clinics, syringe exchange programs or low-threshold housing. The main reasons for this were that this could pose a problem for clients trying to reduce or stop using drugs and that the services would risk criticism from politicians or be visited more frequently by the police.

Several social workers talked about gradually moving boundaries or increasing their acceptance of risky situations and behaviors. This is how Peter, a supervisor at a low-threshold residence reasoned about this risk.

I was lucky enough to work with a Danish [social worker], quite early in my career, who talked a lot about co dependency in terms of us becoming tolerant of overdoses, threats and violence, because we dealt with these matters a lot and we stopped reacting to them. And it became food for thought, so I try, I talk to the staff here about it. That you should be aware of these things.

The quote points to the importance of the staff constantly reflecting on whether boundaries or acceptance are beginning to shift. The quote also highlights the risk of what is referred to as “codependency” and the difficulty in determining when and how to act on clients' vulnerability and risk-taking, which could, for example, involve overdoses, self-harming behavior, deteriorating physical or mental health or living in a very destructive and violent relationship. This points to difficulties setting boundaries due to the need to consider the client’s privacy and autonomy on the one hand, and protection and care on the other. Witnessing people in a very destructive and vulnerable situation was also experienced by some social workers as psychologically stressful.

Stella, who worked at an OST clinic, talked about the difficulty in deciding when to intervene and make a report of concern and suggest compulsory care:

If we see that they have been in the hospital, had overdoses or infected injection wounds… And they don’t take care of themselves, if we don't see any positive change, they just keep falling, falling, falling…. Yes, when we are really worried about their lives and health. You could say that is the limit.

Other social workers were of the same opinion and believed that the acceptance must generally be high in harm reduction services, that the general rule must be to respect the person's autonomy and integrity, but that you have an obligation to intervene in life-threatening situations, or when you see that the individual is on a path toward increasingly poor physical and mental health.

## Discussion

Social work with PWUD has traditionally focused on rehabilitation, with abstinence as a primary goal. This can be something positive if the work is in line with the client’s goals but can also be problematic if it is perceived as forced or intruding. In recent years, harm reduction has gained an increasingly more important role in the work with PWUD, and social workers are key professionals in many low-threshold facilities and harm reduction services for PWUD in Sweden, as well as in other countries. This raises questions about how social workers understand the concept of harm reduction in relation to rehabilitation.

Social workers are important professionals for PWUD as they influence both what interventions are provided and how they are carried out [32, 36]. Social workers have a large degree of discretion in their work, and they can thus act as both gatekeepers, hindering the development of harm reduction, and as pioneers breaking new ground. The social workers we interviewed had a very positive attitude toward a harm reduction perspective and saw this as a natural part of social work with PWUD. Some had themselves shown “moral courage” [37] by pushing for further development of harm reduction in the region, by criticizing existing zero-tolerance policies, or by introducing new harm reduction initiatives despite opposition. This is interesting since, historically, social workers in Sweden have opposed the development of important harm reduction services. A recent survey study from three different regions of Sweden also showed that the social workers generally had positive attitudes toward harm reduction, which, in line with our study, indicates that there has been a change in attitudes over time [30].

Scholars have argued that a harm reduction perspective in social work with PWUD could imply a reduced focus on abstinence, lowering thresholds, and increasing equality in the relationship between client and professional [1, 3, 6–8]. Several of these advantages were also highlighted by our interviewees, not least a more honest, genuine relationship with clients and an opportunity to focus on goals other than abstinence. There seems to be a great deal of agreement about the benefits of a harm reduction perspective within social work with PWUD. However, there is a lack of discussion about what a social work perspective could contribute to harm reduction services. In line with Vakharia and Little [3], we argue that social workers can have important or leading roles in many harm reduction services [3]. Based on the accounts of the social workers interviewed, we use the concept of Harm Reduction Social Work (HRSW, in Swedish: *skadereducerande socialt arbete*) to show how social work and harm reduction, through a number of common principles and strategies, can be combined to broaden and improve services for PWUD.

In doing HRSW the social workers expressed the importance of having low thresholds for services and of “meeting the client where they are at”, without demands for lifestyle change or abstinence. They pointed to the importance of primarily focusing on saving lives and reducing risks and vulnerability. At the same time, HRSW involved a holistic perspective on the individual’s life situation and opportunities that can make a new scope of actions visible to the client. The social workers talked about the importance of strengthening the individual’s resources, skills, and networks. This is in line with the concept of recovery capital and a person-in-environment approach, where focus is put on helping individuals to develop their social, physical, human, and cultural capital to enhance quality of life and gain control over drug use [15, 38].

Social workers are trained to see the individual in a larger context and to understand and navigate society’s various support systems and bureaucratic processes, something the interviewed social workers talked about in terms of being “the spider in the web” and “building bridges”. This can create an opportunity to guide and support clients in their contacts with authorities and safeguard their social and human rights [39]. Several social workers also highlighted the importance of a good working alliance, focusing on the individuals’ strengths, skills, and goals, as well as enabling enjoyment and pleasure. This focus has similarities with strengths-based approaches or strengths-based case management. This approach has been described as central in social work practice and as particularly important in the work with marginalized people with mental health and/or drug use problems. Harm reduction services have been pointed out as one central domain for strength-based approaches [40]. The holistic perspective that characterizes social work speaks for the importance of including social workers in most harm reduction services.

In doing HRSW the social workers recognized the importance of helping clients to improve their life situation and to facilitate motivation and change, as long as this is in line with the clients’ goals. They also argued for that harm reduction in many situations is a prerequisite for, rather than a counterpoint to, rehabilitation, recovery or abstinence. More recent conceptualizations of recovery acknowledge that abstinence does not have to be a final goal and that recovery can take place within the context of continued drug use [14]. This is in line with the perspective of many of the interviewed social workers, indicating that social workers are professionals who have the right competence to work with recovery and rehabilitation within a harm reduction framework.

Some of the interviewed social workers referred to the holistic perspective and motivational work as doing *“more than harm reduction”*. This focus can have several explanations. The social workers seemed to view motivational work as crucial both in relation to the target group's situation and needs and in relation to their own professional competence and ethics. They also saw a need to broaden the focus and range of services within certain harm reduction services where health-related goals were being prioritized too strongly in relation to goals such as social integration and rehabilitation [11]. Allowing for harm reduction services to also incorporate strategies to facilitate rehabilitation and abstinence has been suggested on the basis that many PWUD that reach out to harm reduction services hope to achieve lifelong abstinence [41]. The social workers’ focus on motivational work and change can also be interpreted as a way to legitimize activities that are not in line with the zero-tolerance drug policy model or social workers’ overall statutory mission to help PWUD to become abstinent and reintegrated into society [20]. The idea of doing more than harm reduction, for instance in terms of motivational work, can generally be seen as something positive, but it could also pose a risk of clients ending the contact if they perceive this work as coercive or intruding.

Based on our interviews, it is clear that harm reduction as a starting point is far from obvious for all social workers or managers. This has also been discussed in other contexts, such as Canada [36]. As some interviewees pointed out, harm reduction must not become the only solution or out-compete other efforts such as prevention, in-patient treatment or housing first; there must also be room for social work with other goals and perspectives. Many help-seeking PWUD have abstinence as a primary goal and demand treatment and services with this orientation. Some social institutions or referral agents, such as probation services, child protection services, work training programs, etc., may mandate abstinence-only treatment and requirements for control [3]. Although some harm reduction principles and strategies can be useful in all types of social work, the term HRSW can be used to describe social work in harm reductions settings or social work with a clear focus on reducing harm, especially in contexts such as Sweden, where social work traditionally has had clear focus on abstinence.

The fact that harm reduction and recovery are contested and ambiguous concepts points to the importance of discussing the meanings of and relationship between these in practical social work, as well as in social work education. This can reduce the risk of reproducing simplistic notions of the concepts and show how it is possible to combine principles of harm reduction and recovery in social work with PWUD. How social workers conceptualize substance use problems, rehabilitation and recovery, will affect the types of interventions that they suggest or provide, including harm reduction services [42]. Scholars in social work have argued for the need to include topics such as substance use problems and harm reduction to a greater extent within the curriculum of social work education, as doing so can enhance students’ knowledge and prepare them to practice social work with PWUD in a more pragmatic and humanistic way [2, 43]. Discussing some of the challenges that the social workers faced in doing HRSW could also provide a good opportunity for social work students to prepare for dealing with dilemmas in social work practice with vulnerable PWUD.

Doing HRSW has its challenges, which can vary depending on the local context. The social workers defined challenges on both the macro and the micro levels, but also described strategies to deal with some of them. Macro level challenges concerned national legislation and guidelines hindering harm reduction, and negative attitudes to or lack of knowledge of harm reduction among professionals and senior officials. The social workers illustrated how they could act as agents of change by arguing for the importance of harm reduction to managers or by introducing small-scale efforts or strategies with a harm reduction focus. This might in the long term also influence changes at the policy level.

On a micro level, challenges were, for example, about the lack of legitimacy of social workers in performing certain medical tasks, and about difficulties in setting boundaries and making trade-offs between the client’s privacy and autonomy on the one hand and protection, care, and motivational work on the other. Other challenges concerned not accepting or normalizing violence, life-threatening behaviors, and not becoming “codependent” with one’s clients. Although a contested concept, the notion of codependency was developed within the 12-step movement and is usually used to describe problematic behaviors of spouses or relatives of people who use drugs, such as an extreme focus on others’ needs, being self-sacrificing, and adopting dysfunctional coping aimed at preventing conflict [44]. The concept seems useful for some social workers in describing strategies to handle boundaries toward clients in their professional work. The use of the concept by the interviewees suggests that they try to balance the need for empathy and closeness to their clients with keeping a professional sense of distance. Discussing “codependency” and boundaries may be a strategy used by social workers to engage in self-care practices to reduce stress and enhance well-being [45]. This might be particularly important in low threshold harm reduction services, where clients experience high degrees of vulnerability.

The social workers generally saw their work as important, rewarding, and fun. Some social workers however struggled to find strategies to cope with the psychological stress of witnessing long-term suffering and destructive behaviors, something that has also been discussed in a study of ‘wet’ eldercare facilities in Nordic countries [46]. Accepting destructive or risky behaviors may, in addition to constituting a moral challenge, also clash with policies of social work, since Swedish social services are generally regarded as having a moral and legal imperative to act if witnessing or suspecting self-destructive or life-threatening behavior [46].

Some of the challenges outlined above might be reduced with increased professional supervision, introducing methods or guidelines for how to deal with threats and conflicts, as well as a continuous dialog within the workgroup about how different dilemmas should best be handled and boundaries drawn. The problem with, on the one hand, a medically oriented harm reduction work, and on the other hand, limitations in social workers’ medical competence or legitimacy, speaks for the importance of multi-professional teams in the work with PWUD. Since drug addiction usually includes biological, psychological, and social aspects, these teams should ideally consist of doctors/nurses, psychologists, and social workers. A scoping review of stakeholder preferences for supervised consumption site designs showed that both PWUD and stakeholders recommended these sites to be integrated within or near other social and health services and argued for a broad spectrum of services and staff with different competencies and backgrounds [47].

This is a first suggestion for the concept of HRSW. The concept needs to be discussed, developed, and adapted based on different contexts. HRSW can, as shown in this study, be particularly relevant for services targeting PWUD with a high vulnerability, but it could also be applied to social work more generally. This study has focused on professional social workers, but important HRSW is also carried out by non-professionals, by voluntary organizations, and by PWUD. HRSW can be a starting point or perspective in the work with PWUD or with people who engage in other risky behaviors, regardless of professional affiliation or background. Furthermore, social work with PWUD in Sweden can differ significantly from that carried out in other countries. Even Scania, the region in Sweden in which the study was conducted, differs in certain respects in relation to other regions in the country. The context-specific aspects are important to consider in the interpretation and possible generalizability of the results. Further research is needed on how social workers in harm reduction services in countries with different legislation, drug policies, or social work organization understand the concept of harm reduction and how they deal with the various dilemmas and challenges in everyday social work.

## Conclusions

Social workers are key professionals in services for PWUD. We use the concept of Harm Reduction Social Work to show how social work with PWUD can have a primary focus on reducing harm and risks, while at the same time facilitate motivation, change and recovery. Social workers can contribute with a holistic perspective on clients’ resources and needs and bridge the gap between services focused on harm reduction on the one hand and abstinence on the other hand. Concepts such as harm reduction and recovery need to be discussed in social work education, and the suggested principles of HRSW can provide guidance in practical social work with PWUD.

## Data Availability

The datasets generated and/or analyzed during the current study are not publicly available due to protection of informants’ privacy but are available from the corresponding author on reasonable request.
